# Exosomal non coding RNAs as a novel target for diabetes mellitus and its complications

**DOI:** 10.1016/j.ncrna.2023.02.001

**Published:** 2023-02-07

**Authors:** Albert Sufianov, Andrey Kostin, Sema Begliarzade, Valentin Kudriashov, Tatiana Ilyasova, Yanchao Liang, Albert Mukhamedzyanov, Ozal Beylerli

**Affiliations:** a Educational and Scientific Institute of Neurosurgery, Рeoples’ Friendship University of Russia (RUDN University), Moscow, Russia; b Department of Neurosurgery, Sechenov First Moscow State Medical University (Sechenov University), Moscow, Russia; c Research and Educational Resource Center for Immunophenotyping, Digital Spatial Profiling and Ultrastructural Analysis Innovative Technologies, Peoples' Friendship University of Russia, Moscow, Russia; d Republican Clinical Perinatal Center, Ufa, Republic of Bashkortostan, 450106, Russia; e Gastric Cancer Center, West China Hospital of Sichuan University, China; f Department of Internal Diseases, Bashkir State Medical University, Ufa, Republic of Bashkortostan, 450008, Russia; g Department of Neurosurgery, The First Affiliated Hospital of Harbin Medical University, Harbin, 150001, China; h City Clinical Hospital №21, Ufa, Republic of Bashkortostan, 450071, Russia

**Keywords:** Exosomes, Non-coding RNAs, Diabetes mellitus, Pathogenesis, Therapy, Diagnostics

## Abstract

Diabetes mellitus (DM) is a first-line priority among the problems facing medical science and public health in almost all countries of the world. The main problem of DM is the high incidence of damage to the cardiovascular system, which in turn leads to diseases such as myocardial infarction, stroke, gangrene of the lower extremities, blindness and chronic renal failure. As a result, the study of the molecular genetic mechanisms of the pathogenesis of DM is of critical importance for the development of new diagnostic and therapeutic strategies. Molecular genetic aspects of the etiology and pathogenesis of diabetes mellitus are intensively studied in well-known laboratories around the world. One of the strategies in this direction is to study the role of exosomes in the pathogenesis of DM. Exosomes are microscopic extracellular vesicles with a diameter of 30–100 nm, released into the intercellular space by cells of various tissues and organs. The content of exosomes depends on the cell type and includes mRNA, non-coding RNAs, DNA, and so on. Non-coding RNAs, a group of RNAs with limited transcriptional activity, have been discovered to play a significant role in regulating gene expression through epigenetic and posttranscriptional modulation, such as silencing of messenger RNA. One of the problems of usage exosomes in DM is the identification of the cellular origin of exosomes and the standardization of protocols for molecular genetic studies in clinical laboratories. In addition, the question of the target orientation of exosomes and their targeted activity requires additional study. Solving these and other problems will make it possible to use exosomes for the diagnosis and delivery of drugs directly to target cells in DM. This study presents an analysis of literature data on the role of exosomes and ncRNAs in the development and progression of DM, as well as the prospects for the use of exosomes in clinical practice in this disease.

## 1 Introduction

Exosomes (exosomes) secreted outside cells are "star" molecules in extracellular vesicles, they can carry and transfer a variety of biological signals, and act on distant cells through autocrine, paracrine, etc. In order to achieve the purpose of information transmission between cells. At present, the latest research has introduced exosomes into the field of diabetes, which has opened up a new situation for research in this field [1]. The content of exosomes depends on the cell type and includes proteins such as annexins, tetraspanins, major histocompatibility complex molecules, cytoskeletal proteins, enzymes, and signaling proteins, as well as mRNA, DNA and non-coding RNAs. NcRNAs, which make up over 98% of human genome expression products, are a group of RNA molecules that have little transcriptional value but play a significant role in gene expression regulation through epigenetic and posttranscriptional mechanisms [2]. This includes microRNAs (miRNAs) with a length of 19–25 nucleotides, long non-coding RNAs (lncRNAs) with over 200 nucleotides, and ring-loaded RNAs (circRNAs) that have a closed loop structure. While these ncRNAs do not produce proteins, they still play an important role in various physiological activities through their regulation of target genes. MiRNAs bind to messenger RNAs (mRNAs) that have a complementary 3′-untranslated region (3′-UTR), while lncRNAs have non-random short open reading frames (sORFs) and are involved in chromatin modification, chromosome recycling, and DNA transcription processes. Over the past few years, it has become clear that non-coding RNAs (ncRNAs) play a crucial role in maintaining normal body functions and that their abnormal expression is closely linked to the development of numerous diseases, including diabetes mellitus (DM) [[3], [4], [5]]. An increasing number of studies have centered on ncRNAs in DM and its associated complications, suggesting that ncRNAs can interact with insulin [6]. There is also evidence to indicate that ncRNAs may serve as modulators and markers of diabetic cardiovascular disease [[7], [8], [9], [10]]. This article reviews the role of exosomes and ncRNAs as part of they in the occurrence and development of diabetes and its complications, and discusses the role and prospect of exosomes and ncRNAs as a target for diabetes treatment and in the diagnosis and treatment of diabetes. This article summarizes the origin and classification of extracellular vesicles, focuses on summarizing the imaging methods in the process of extracellular vesicle origin, isolation, dynamic uptake and release, discusses the advantages and disadvantages of different methods, and provides a basis for the study of extracellular vesicles.

## 2 Biological characteristics and functions of exosomes

Exosomes are nanosized extracellular vesicle-like bodies with a membrane structure secreted by cells. The diameter of exosomes is 30–150 nm, and the density is 1.10–1.19 g/ml [11,12]. There are about 1014 exosomes in the human body, which is close to 1000–101000 per cell on average. Exosomes, as a type of membrane vesicles, mainly consist of intracellular multivesicular bodies that fuse with the cell membrane and are released into the extracellular matrix. Under an electron microscope, exosomes look like flattened spheres wrapped in lipid bilayers, with a characteristic glass holder shape [13]. Exosomes can produce almost all eukaryotic cells, including some microorganisms [[14], [15], [16]]. Exosomes can stably exist in extracellular fluid, including cell culture supernatant, plasma, serum, saliva, urine, amniotic fluid, ascites, milk, cerebrospinal fluid, nasal lavage fluid, joint cavity fluid, semen, prostate fluid, bile and other biological fluids. Cells that secrete exosomes may include T cells, B cells, platelets, dendritic cells, mast cells, etc. In fact, in addition to exosomes, extracellular vesicles also include microvesicles (MVs) and apoptotic bodies. Their diameter is larger than that of exosomes, and the mechanism also differs from that of exosomes [17]. As for the diameter of exosomes, in fact, different types of cells will also determine the size of exosomes, for example, the diameter of exosomes secreted by adipocytes is relatively large, about 150–200 nm [18]. Therefore, the exosome diameter cannot be absolutely limited to <150 nm. In addition, only exosomes can be continuously released by cells, while other extracellular vesicles can only be released by activated or apoptotic cells, which is also a difference between exosomes [19].

**Fig. 1:**
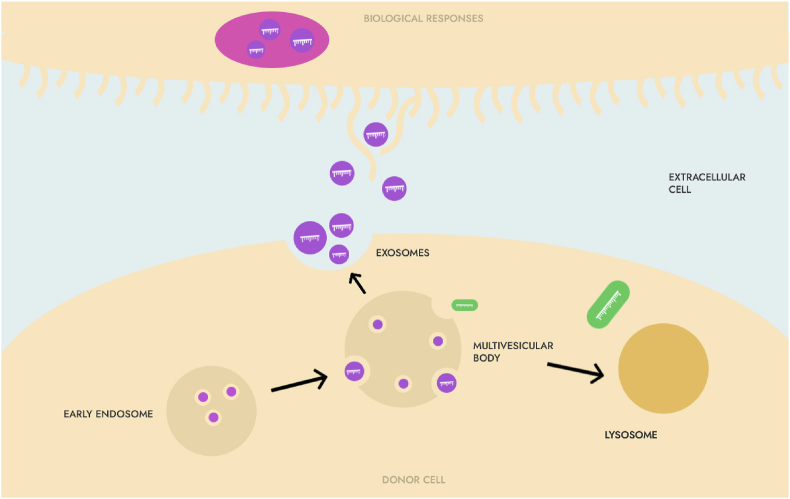
Exosomes-mediated intercellular transmission.

The formation of exosomes begins with endocytosis of extracellular substances or membrane proteins to form small vesicles, which then fuse with each other to form early endosomes (early endosomes, EE), and then EE are gradually transported through intracellular transport and become late endosomes (late endosomes, LE). At this time, the endosome membrane buds inward through reflex folds, and sorted DNA fragments, circular RNA (cirRNA), mRNA, microRNA (microRNA) in the cell are, miRNA), proteins, transcription factors, etc. are packaged, forming multiple intraluminal vesicles (intraluminal vesicles, ILVs), which are precursors of exosomes [19]. LEs contain multiple luminal vesicles called multivesicular bodies (MVBs) that are responsible for the transport and sorting of proteins. Then, some MVBs fuse with the cytoplasmic membrane and release exosomes into the extracellular matrix, while other MVBs fuse with lysosomes, and their content degrades and participates in recycling [19]. Exosomes released into the extracellular matrix re-enter the recipient cells via endocytosis or by recognizing specific receptors on the membrane surface and release the "cargo" they carry into the cytoplasm of the recipient cells to play a role in signal transduction. The composition of exosomes and the type of recipient cells determine the function of exosomes (Fig. 1).

Exosomes can carry and transport many biological molecules, such as DNA fragments, circRNA, mRNA, miRNA, functional proteins, transcription factors, etc., to achieve the purpose of intercellular information transfer; and their own membrane structure can also express various antigens, antibody molecules, thus participating in the exchange of information and substances between cells, play an important role in many physiological and pathological processes: such as cell communication, cell migration, differentiation, angiogenesis, immune response, antigen presentation, tumor invasion, etc., and can also be used as nanocarriers for loading genes or drugs to reach target organs, etc. [20].

## 3 The importance of exosomes in diabetes research

Exosomes play an important role in insulin sensitivity, glucose homeostasis, and vascular endothelial function [21]. Diabetes mellitus is a common metabolic disorder characterized by dysfunction of insulin secretion by pancreatic β cells, varying degrees of insulin resistance combined with relative lack of insulin. Organs such as the pancreas, liver, muscle or fat are involved, and the communication between these organs is a key link in maintaining glucose homeostasis [20]. When a patient develops metabolic disorders, the number of exosomes in the circulating blood increases [22]. The more severe the insulin resistance, the more dysfunctional the islet β cells are, the more changes in insulin signaling proteins in exosomes are, and these exosomes are preferentially absorbed by white blood cells. Internalization, changing the function of white blood cells [23], exosomes also contain components that can cause vascular dysfunction [22]. The relationship between diabetes and exosomes has received extensive attention. Studies have shown that exosomes are involved in the occurrence and development of diabetes and its related complications. It can not only be used as a biological marker for early diagnosis and staging of diabetes, but also as a target for diabetes treatment, and more importantly, it can Monitor the response of diabetic patients to treatment, and provide a basis for implementing individualized treatment of diabetes [20,24].

### 3.1 Relationship between exosomes and insulin and its receptors

Pancreatic β-cells are the only source of insulin. Insulin, a protein hormone secreted by pancreatic β-cells in response to endogenous or exogenous stimuli, is involved in the regulation of glucose metabolism and control of blood sugar balance [25]. Insulin-secreting β-cells and insulin-responsive tissues release exosomes containing proteins and microRNAs extracellularly and can be transferred to other metabolic organs or immune endothelial cells. Exosomes function autocrine and paracrine, which contributes to the maintenance of glucose homeostasis or induces insulin resistance [26]. The number of exosomes in peripheral blood or adipocyte-derived exosomes positively correlated with the insulin resistance index (HOMA-IR) to assess the homeostasis model, also associated with insulin sensitivity [27,28]. Exosomes regulate insulin sensitivity in at least two ways: one is by regulating the inflammatory pathway; the other is through direct interaction with insulin-sensitive organs, which can directly or indirectly affect the insulin signaling pathway.

Insulin binds to the insulin receptor (IR) on the cell membrane and activates tyrosine kinase, thereby initiating the intracellular insulin signaling pathway. Calpain-2 is a calcium-dependent protease that does not contain a signal peptide, but is an enzyme necessary for the regulation of the secretory pathway, and its role is not to degrade proteins, but to split them. Under high glucose conditions, exosomes secreted by cells contain calpain2, which allows calpain 2 to be released into the extracellular space with the help of exosomes; The site directly catalyzes the splitting of IR to become a soluble insulin receptor (sIR); the next step is to initiate the splitting of IR in the cell membrane by γ-secretase. The continuous division of IR in the extracellular part and the inner part of the cell membrane eventually leads to the inhibition of tyrosine phosphorylation of insulin receptor substrate-1 (insulin receptor substrate1, IRS-1) and protein kinase B (Akt) phosphorylation, and then Impairs the insulin signaling pathway, leading to insulin resistance. This is why plasma sIR levels in patients with type 2diabetes mellitus (T2DM) are negatively correlated with insulin sensitivity. Metformin may inhibit the release of calpain 2 from exosomes, interfere with IR splitting (through the pathway of knocking out calpain 2 and γ-secretase), restore the functions of IRS-1 and Akt, and re-establish insulin signaling, thereby alleviating insulin resistance, enhance insulin sensitivity. Therefore, the study of exosomes actually discovered a new mechanism of insulin resistance [29].

### 3.2 Exosomes and adipose tissue-associated insulin resistance

Adipose tissue is a dynamic endocrine organ, which secretes a variety of adipokines, enzymes, growth factors and hormones that regulate glucose and lipid metabolism to regulate the homeostasis of whole body energy [[30], [31], [32]]. Insulin resistance is partly related to substances secreted by fat cells. In insulin-resistant obese people with prediabetes, it was found that their adipocytes can produce exosomes, which are called adiposomes (ADEs) [33]. Lipid raft microdomains containing glycosylphosphatidylinositol-anchored protein in ADEs can be used for phosphodiesterase and 5′-nucleotidase CD73 to hydrolyze cAMP in the cell, thereby blocking the biochemical action transmitted by cAMP. ADEs are secreted by larger donor adipocytes and subsequently phagocytized by smaller adipocytes, accompanied by accelerated fatty acid esterification of triglycerides and slower release of triglycerides [33]. Angiogenic factors produced by adipose tissue are loaded into ADEs and participate in angiogenesis; ADEs are also involved in inducing cell migration and lumen formation in human umbilical vein endothelial cells. Exosomes released by endothelial cells of adipose tissue are rich in plasma components. In this way, substances in plasma can be transferred to adipocytes, and biological information about changes in the nutritional status of the whole body can be transmitted to adipocytes. This fully demonstrates that through exosomes The importance of communication to maintain metabolic balance [34]. ADEs negative for CD14 expression have been found to be inversely associated with T2DM risk [35].

ADEs contain approximately 7000 mRNAs and 140 miRNAs, most of which are transcripts of adipocyte-specific and dominant genes, whose abundance is mainly related to donor cells, and ADEs deliver RNAs in a paracrine or endocrine manner [36]. The miRNA expression profile contained in the ADEs of pre-diabetic insulin-resistant obese people has changed significantly compared with healthy people, and can further regulate recipient β cells by transmitting differentially expressed miRNAs, and even regulate distant organs (such as liver) intracellular gene expression [37,38]. In the liver, fibroblast growth factor 21 (FGF21) acts to reduce miRNA, and if the level of mature miRNA in the tissue is significantly reduced, the level of mature miRNA in circulating exosomes is also significantly reduced [39]. Transplantation of both white and brown adipose tissue into the tissue can partially restore circulating miRNA levels by reducing FGF21 and improve glucose tolerance [39]. Dicer enzyme, which is used to process miRNA, is downregulated in adipose tissue with age [30]. Knockout of fat-specific Dicer enzymes hypersensitizes mice to oxidative stress. Therefore, an imbalance of miRNA regulation in adipose tissue will directly lead to an increased incidence of metabolic diseases such as diabetes in the elderly. Importantly, these effects may not simply be due to problems in the fat cells themselves, but also represent a defect in their communication with other organs [39,40]. Skeletal muscle cells can take up ADEs, and miR-27a secreted by ADEs can inhibit the target gene peroxisome proliferator-activated receptor-γ (PPARγ), causing impaired glucose uptake in skeletal muscle [41].

In addition to miRNAs, following a diet or bariatric surgery significantly increased the concentration of long non-coding RNA (long non-coding RNA, lncRNA) contained in exosomes derived from stem cells of the peritoneal omental adipose tissue of obese people [42]. After that, the amount of lncRNA significantly decreased [43].

ADEs are also rich in adiponectin, an adipokine secreted only by adipocytes, and they are involved in lipid metabolism and insulin resistance [[44], [45], [46]]. Exosomes rich in adiponectin can influence the metabolism of distant cells. Fatty acid-binding protein (aP2) can regulate intracellular lipid transport in various tissues in non-classical ways upon lipase activation, and the level of aP2 in exosomes increases significantly upon stimulation of lipolysis. In obesity, adipose tissue becomes resistant to insulin-mediated inhibition of lipolysis, thereby increasing aP2 secretion, which leads to increased hepatic glucose output and diabetes [47]. In addition, Sonic Hedgehog (Shh) is also a type of exosome-wrapped protein. Shh expression in circulating exosomes in T2DM patients is elevated, which may stimulate macrophages to secrete inflammatory factors and mediate M1 through the Ptch/PI3K signaling pathway. Polarization of macrophages, which in turn leads to insulin resistance of adipocytes [48]. Exosomes also contain some adipocyte-specific proteins such as tumor necrosis factor α (tumor necrosis factor α, TNF-α), macrophage colony stimulating factor (macrophage colony stimulating factor, MCSF), and retinol-binding protein 4 (RBP -4) and so on. ADEs in patients with gestational diabetes are selectively enriched in a specific set of proteins associated with changes in glucose metabolism in placental cells [49].

Interestingly, after culturing human adipose tissue in vitro, subcultured adipose tissue sections were transferred to a new medium, and these human adipose tissue explants released exosomes, the content of which depended on the activation of adipokines. induced AKT phosphorylation in hepatocytes [50]. Exosomes isolated from the culture medium of human adipose tissue cells kept under hypoxic conditions directly affect other functions of adipocytes, reducing glucose uptake and insulin-mediated AKT phosphorylation [51].

Exosomes released from adipose tissue mesenchymal stem cells (AT-MSCs) treated obese mice by transmitting and activating signal transducer and activator of transcription 3 (STAT3), Guided M2-type replacement of activated macrophages, improved insulin sensitivity, reduced obesity, and improved hepatic steatosis [52]. Also MSCs, exosomes released from aged mouse marrow mesenchymal stem cells (M-MSCs) composed of adipocytes, muscle cells, and liver cells can induce insulin resistance in vivo and in vitro, the amount of miR-29b-3p was significantly increased in exosomes released from M-MSCs, downregulation of miR-29b-3p can significantly improve insulin resistance [53]. In conclusion, exosomes can serve as a new target for the treatment of insulin resistance in obese people [54].

### 3.3 Exosomes and low-grade chronic inflammation and insulin resistance

Type 1 diabetes mellitus (T1DM) and T2DM have different pathogenesis: T1DM is due to the gradual loss of insulin-producing cells, resulting in low or no insulin production; insulin resistance. Although the pathogenesis of T1DM and T2DM are quite different, their causative factors, course, pathophysiology, disease progression, and complications are linked: both are caused by a combination of genetic susceptibility and environmental factors, and only susceptibility genes Different; T2DM can start with ketoacidosis, while some patients with T1DM have an insidious onset, especially adult patients with positive islet autoantibodies; Obesity and insulin resistance develop progressively; both involve interactions between the immune and metabolic systems [55]. Notably, chronic inflammation is a common feature of both types of diabetes [56,57]. Recently, exosomes have been identified as intermediates linking inflammation and diabetes [58]: Islet mesenchymal stem cells (MSCs) from autoimmune susceptible animals release highly pro-inflammatory exosomes that contribute to T1DM occurs [59]; low-grade inflammatory exosomes in obesity may stimulate the release of pro-inflammatory exosomes, accelerate T2DM [60,61], and eventually lead to systemic insulin resistance [27]. Therefore, obesity is the main pathogenic factor leading to insulin resistance. When pancreatic β cells cannot secrete enough insulin to compensate for the decrease in insulin sensitivity, it can lead to the occurrence of T2DM [62].

### 3.4 Exosomal miRNAs and diabetes

Among the various contents contained in exosomes, siRNA is a very common type of non-coding RNA (about 19–22 nucleotides long) consisting of a single hairpin about 70–90 bases in size. processed by the Dicer enzyme. They bind to mRNAs, targeting them, causing their degradation or repressing their translation [63]. Studies have shown that the concentration of microRNA in exosomes is always higher than in body fluids, and its copy number is sufficient to exert a biological effect on recipient cells [64,65]. miRNAs secreted by exosomes can be transferred to neighboring cells, exposing mother cells to the pathophysiological environment characteristic of diabetes, can regulate the release of miRNAs and influence the survival of recipient cells, a new mechanism of intercellular communication regulates the activity of pancreatic β-cells [66].

Studies have shown that exosomes secreted by the pancreas contain miP-375, which can regulate insulin secretion and islet formation. miR-375 is one of the few miRNAs with demonstrated tissue specificity isolated from serum or plasma [67,68]. Deficiency of miR-375 leads to hyperglycemia, accompanied by increased secretion of pancreatic α-cells, increased gluconeogenesis, and increased production of hepatic glycogen [69]. Overexpression of miR-375 inhibits insulin secretion. In-depth studies have shown that myotrophin (MTPN) is the target of miR-375 [70]. Exosomes released from adipose-derived macrophages lead to glucose intolerance and insulin resistance, and these exosomes target PPARγ, which is highly expressed in adipocytes, to reduce insulin sensitivity of other tissues (e.g., the liver) [71].

Cell populations derived from peripheral blood mononuclear cells (PBMC) can promote angiogenesis, and miRNAs that regulate angiogenesis are key regulators. miR-126 of the CD34^+^ PBMC subpopulation is highly expressed in vascular endothelial cells. The release of exosomes can lead to impaired angiogenesis. Altered expression of iR-126 in CD34^+^ PBMCs from diabetic patients resulted in impaired pro-angiogenic effects [72]. Some studies summarized many miRNAs in exosomes related to diabetes process: miR-27a-3p, miR-27b-3p, miR-192 related to glucose intolerance; miR-122 related to disease progression; let-7a, let-7f, which reflect blood sugar control and post-medication response. These exosomal miRNAs play a key role in the regulation of glucose and lipid metabolism [73].

However, since miRNA may be involved in a variety of physiological and pathological processes, it is difficult to identify a certain miRNA as a specific biological marker, which also limits the clinical use of miRNA. However, after continuously expanding the sample size and summarizing laboratory or clinical data in the future, it is just around the corner to find specific miRNA biomarkers [20].

We would also like to add a few words about miRNAs, whose relationship with exosomes has not yet been proven, but which have been proven to affect the development of DM. Studies have shown that increasing the expression of certain miRNAs such as miRNA-494, miRNA-92a, miR-136–5p, and miR-149–5p can improve pancreatic β-cell proliferation and insulin secretion [[74], [75], [76]]. Other miRNAs, such as miR-150–3p and miR-17–5p, can protect β-cell function by inhibiting focal death and activating PDX1 signaling, respectively [77,78]. miRNA-16–5p, expressed at lower levels in type 1 diabetes mellitus (T1DM) patients, can inhibit high-glucose-induced pancreatic β-cell apoptosis by targeting CXCL10 [79]. The downregulation of miR183–3p has been found to treat gestational diabetes mellitus (GDM) by reducing skeletal muscle insulin resistance (IR) [80]. The exosome-derived miR-26a increases insulin sensitivity by enhancing insulin signaling and reducing hyperinsulinemia, a typical symptom of type 2 diabetes mellitus (T2DM) [81]. The miR-17-5p-Mfn1/2-NF-ΚB pathway has anti-inflammatory and anti-apoptotic effects in GDM [82]. miR-1249–3p has reduced IR and inflammation in a mouse model of T2DM [83]. miRNA-26a promotes regulatory T cells to suppress T1DM [84]. The miR-212/132-enriched extracellular vesicles can be used to differentiate induced pluripotent stem cells into pancreatic β-cells, enabling cell replacement therapy for T1DM [85].

### 3.5 Other noncoding RNAs in exosomes and their roles in diabetes

Other types of noncoding RNAs such as circular RNA (circRNAs), P-element induced Wimpy testis (PIWI)-interacting RNAs (piRNAs), and long noncoding RNA (lncRNAs) have also been found to be involved in the pathological process of diabetes. CircRNAs are a form of noncoding RNA that form a circular and continuous loop, making them resistant to degradation and more stable compared to linear RNAs. Studies have shown that circRNAs often regulate miRNA-target gene transcription and act as miRNA sponges. Some circRNAs have been linked to the progression of diabetes, including reducing β-cell proliferation, decreasing survival, and impacting insulin secretion [[86], [87], [88]]. PiRNAs are another class of small RNAs found in germline cells, playing a role in spermatogenesis and regulating gene expression. They have been found to be expressed in pancreatic islets and participate in controlling β-cell activities [89]. Although both circRNAs and piRNAs exist in exosomes, there is still limited evidence on changes of exosomal circular RNA or piRNA in diabetic state.

LncRNA is a type of noncoding RNA that is longer than 200 nucleotides. It affects gene expression through epigenetic, transcriptional, and posttranscriptional mechanisms. Several lncRNAs, such as ANRIL, H19, MALAT1, Sox2OT, and MEG3, have been implicated in the pathology of diabetes, specifically type 2 diabetes [[90], [91], [92], [93], [94]]. Although there is limited research on the connection between exosomal lncRNAs and diabetes, a study found that lncRNA-p3134 was increased four-fold in serum exosomes and correlated with fasting blood glucose and HOMA-β levels. Further study showed that the secretion of lncRNA-p3134 positively regulated insulin secretion by promoting key regulators (Pdx-1, MafA, GLUT2, and Tcf7l2) in beta cells and restoring insulin synthesis and secretion in db/db mice, making lncRNA-p3134 a potential compensatory factor for preserving beta cell function in response to high glucose stimulation [95].

### 3.6 Exosomes and T1DM

After revealing the influence of DM1 and delivery of exosomes, it was found that the serum in DM1 contains more exosomes, and miRNAs wrapped in them are involved in the regulation of heart development, and these exosomes can be inherited in fetal mice, which directly leads to an increase in the incidence of congenital heart disease [96].

The exosomes contain potent immunostimulatory substances, and the exosomes released by insulinoma can stimulate an autoimmune response in non-obese diabetes (NOD) mice and islets of Langerhans in NOD mice) and are cultured in vitro, the cultured islet cells release fibroblasts. -like rapidly replicating cells expressing MSC markers including CD105 and stem cell antigen-1. These is let MSC-like cells release highly immunostimulatory exosomes that can activate autoimmune B and T cells in NOD mice. This indicates that exosomes carry the NOD mouse autoantigen, have strong immune activity, and may be a trigger for autoimmunity in diabetic NOD mice [34].

MiR-21–5p is enriched in exosomes released from T cells or islet cells treated with pro-inflammatory cytokines and is elevated in the serum of both T1DM patients and NOD mice, which in turn triggers apoptosis in recipient cells pathway, indicating that this process may play a role in the development of T1DM, and exosomal miR-21–5p may become a T1DM biomarker [97,98].

The production of exogenous interferon-γ in the serum exosome level of prediabetic patients was positively correlated with the disease progression, and CD105+ cells were confined to the peripheral area of normal islet cells, but with the infiltration of lymphocytes, CD105+ cells entered the central area of islets (mainly β cell area), exosome immunization promotes the expansion of diabetic metastatic T cells and accelerates the destruction of islet cells mediated by effector T cells [34]. Exosomes isolated from patients with T1DM for many years found that some miRNAs were up-regulated, such as miR-25–3p; some miRNAs were down-regulated, such as miR-16–5p, miR-302d-3p, miR- 378a, miR-570–3p, miR-574–5p, etc. [99]. The above research results provide ideas for finding new targets for the treatment of T1DM.

Islet cell transplantation is an effective treatment for autoimmune DM 1. Exosomes specifically released from transplanted islets into the circulation have potential diagnostic value for distinguishing between recurrent autoimmunity and immune rejection after injury to pancreatic β-cells, indicating that exosomes are biological markers [100].

Exosomes isolated from MSCs have an immunomodulatory effect and can improve islet function by increasing the number of regulatory T cells and their anti-inflammatory products IL-4 and IL-10; therefore, they can be used to treat DM1 [101]. However, some scientist's express different views on this matter: exosomes isolated from M-MSCs can promote bone tissue regeneration, and in patients with DM1 this function is impaired, which indicates that for patients with DM1, autologous bone marrow stem cell transplantation may be ineffective [102].

### 3.7 Exosomes and T2DM

Amylin is stored in the insulin-secreting granules of pancreatic β cells and is co-secreted with insulin. Its serum concentration is about 1/10 of that of insulin; in the pancreas of many T2DM patients, the content of amylin increases [103]. Pancreatic exosomes from normal people can reduce the formation of amylin by peptide clearance, but pancreatic exosomes and serum exosomes from T2DM patients have no similar effect, and the ratio of C-peptide and lipid composition are different from normal people [104].

Exosomes carry important biological information of T2DM pathogenesis. Exosomes and the miRNA carried by them pass through blood from adipose tissue and penetrate into skeletal muscle and liver. The response induced by this inter-tissue migration may directly lead to the disordered intercellular communication in T2DM related to metabolism [71]. Plasma miR-15a is elevated in patients with T2DM, which plays an important role in insulin production in islet β cells and is associated with disease severity. In fact, the increase of miR-15a in the blood is derived from the exosomes secreted by pancreatic β cells. After the increase of miR-15a, it targets Akt3 to cause oxidative stress, which in turn leads to cell apoptosis [105]. Decreased miR-126 or increased miR-192 and miR-193b are all signals of the pre-existence of T2DM, allowing early identification of at-risk subjects [106,107]. All of the above indicate that the miRNA contained in exosomes secreted by islet cells regulates β-cell function in a paracrine manner, and this situation is significantly different between normal people and T2DM patients [108].

n addition, the level of lncRNAp3134 contained in circulating exosomes of T2DM patients is higher than that of non-T2DM patients, and it is related to fasting blood glucose and HOMA-β levels. Further studies have found that lncRNA-p3134 can promote key regulatory factors (Pdx-1, The expression of MafA, GLUT2, Tcf712) positively regulates the function of glucose-stimulated insulin secretion (GSIS), indicating that the regulation of lncRNA-p3134 in pancreatic β cells can maintain blood glucose homeostasis [109].

DM2 is considered to be a chronic, indolent inflammatory disease affecting immune and endothelial cells [110,111]. The sluggish inflammatory process is characterized by the activation of endothelial cells [112,113]. This cellular activation results in endothelial cells and monocytes secreting cytokines and expressing adhesion molecules such as intracellular adhesion molecule type I (ICAM-1), which in turn leads to immune cell adhesion and transport. into the blood. vessel wall [114,115]. Circulating levels of ICAM-1 are elevated in patients with T2DM, which is a marker of endothelial cell activation [116,117]. Endothelial cells and monocytes can release exosomes, these exosomes regulate the function of endothelial cells and monocytes and are involved in the interaction between endothelial cells and immune cells, which contributes to high activation of endothelial cells and monocytes. in response to glucose is critical [[118], [119], [120]]. In a high-glucose environment, the kinetics of exosomes released by endothelial cells is significantly affected, and exosomes are strongly associated with the activation of inflammatory cells in T2DM or diabetes-related cardiovascular complications [121,122].

Exosomes secreted by human mesenchymal stem cells (human umbilical cord MSC-derived exosome, hucMSC-ex) may alleviate T2DM by reversing insulin resistance in peripheral blood and inhibiting β-cell apoptosis. hucMSC-ex restored IRS-1 phosphorylation (tyrosine site) and Akt expression in DM2, stimulated the expression of muscle glucose transporter 4 (glucose transporter 4, GLUT4) and membrane transport, and increased liver glycogen stores to maintain blood glucose homeostasis, indicating that hucMSC-ex may be a treatment option for T2DM [123].

On the other hand, studies have shown that miR-29b exosomal branched-chain amino acids (BCCAs) in dairy produce excess insulin synthesis and BCAA mTOR-dependent insulin resistance when dairy products are consumed for a long time, indicating that exosomes in dairy products are potential promoters of CD2; whereas miR-29b-mediated and miR-148a-mediated repression of secreted protein, acidic and rich in cysteine (SPARC), inhibition of V-Maf muscle aponeurotic fibrosarcoma (MAFB) homologue B oncogene B may impair insulin secretion, increasing endoplasmic reticulum stress and apoptosis β-cells [124].

### 3.8 Exosomes and gestational diabetes mellitus (GDM)

GDM accounts for approximately 9% of pregnancies and is another manifestation of diabetes [125]. Although GDM usually returns to normal after delivery, increased metabolic demands during pregnancy lead to temporary defects in glucose metabolism. As the incidence of obesity increases, the incidence of GDM also gradually increases, which will affect the health of offspring, because embryos will undergo epigenetic changes due to long-term exposure to a disturbed metabolic environment [126]. Several factors, including placental hormone (PH), released by the placenta, have been implicated in the development of insulin resistance and GDM. However, blood pH levels did not correlate well with maternal insulin sensitivity throughout gestation, suggesting the possibility of other unrecognized mechanisms [127].

The placenta releases exosomes into the maternal circulation from the 6th week of gestation. This process is regulated by factors such as oxygen partial pressure and blood glucose concentration, and is related to placental quality and perfusion. Placental exosomes play an important role in normal placental development and maternal immune tolerance. Different pregnancy periods and pregnancy status will affect the concentration of plasma exosomes. With the prolongation of gestational age, the concentration of plasma exosomes gradually increases, and the exosomes isolated from maternal plasma are biologically active in vitro and bind to target cells through endocytosis, which is related to pregnancy complicated with diabetes and preeclampsia [127]. GDM is associated with skeletal muscle insulin resistance and increased levels of circulating placental exosomes [128]. Placental exosomes from these patients encapsulated some specific miRNAs related to skeletal muscle insulin sensitivity, and they were consistently expressed in placenta, circulating exosomes, and skeletal muscle. Placental exosomes from GDM can reduce the migration and glucose uptake rate of primary skeletal muscle cells with normal insulin sensitivity, suggesting that placental exosomes may play a role in normal pregnancy and changes in insulin sensitivity in GDM. While GDM patients have higher levels of circulating exosomes than normal people, GDM is associated with hyperglycemia-induced fetal placental endothelial dysfunction, GDM-derived exosomes can release more pro-inflammatory factors from endothelial cells, they participate in disease progression of endothelial dysfunction in GDM [115,129].

## 4 Mechanism of exosomes involved in target organ damage in diabetes

Diabetes is prone to be complicated by large and medium blood vessels (atherosclerosis) and microvascular lesions (retinopathy, nephropathy, and neuropathy), which can involve damage to important target organs throughout the body, and eventually lead to target organ failure [130]. The research on exosomes also penetrates into all aspects of diabetic complications.

### 4.1 Exosomes and diabetic nephropathy

Diabetic nephropathy is a serious complication of diabetes and a common cause of end-stage renal disease. Exosomes released by glomerular mesangial cells under conditions of high glucose levels cause damage to podocytes in vitro, leading to diabetic nephropathy [131].

Some believe that protein markers of exosomes may more accurately reflect potential changes in patients with diabetic nephropathy than complete urinalysis, and that the contents are wrapped in the membrane structure of exosomes to avoid degradation by proteases, so that test results are more reliable. exactly [132]. The urine exosomes of patients with diabetic nephropathy contain 352 proteins, among which the urinary exosome protein WT1, secreted by renal epithelial cells, can be used as a non-invasive biological marker to predict early diabetic kidney injury [133]; α1-microglobulin/bicunin precursor (α-1-microglobulin/bicunin precursor, AMBP), histone lysine-N-methyltransferase (MLL3), voltage-gated anion channel protein-1 (voltage-gated anion selective channel protein, VDAC1) is a biological indicator, which can be used to predict early abnormalities of diabetic nephropathy [134]; levels of leucine aminopeptidase (LAP) and dipeptidyl peptidase 4 (dipeptidyl peptidase 4, DPP4) are associated with biological parameters closely associated with the severity of diabetic nephropathy [135].

Stable exosomes in the urine and microRNAs contained in them are a sign of the development of diabetic nephropathy and play an important role in pathogenesis [136]. miR-130a, miR-145 and the number of exosomes in exosomes released from glomerular mesangial cells (GMS) positively correlated with glucose concentration [67]. Compared to patients with T2DM, exosomal microRNA in the urine of patients with T2DM nephropathy has abnormal expression of miP-320c, which may influence TGF-β signaling by mediating thrombin-1 (thrombospondin-1, TSP-1). the index is expected to be used as a new candidate marker of T2DM nephropathy to assess T2DM nephropathy [[136], [137], [138]].

MSC-derived exosomes can induce autophagy, significantly improve kidney function, and repair kidney tissue, which is a novel treatment for diabetic nephropathy [139]. Exosomes secreted by urine-derived stem cells (USCs) can protect podocytes in a high glucose environment, inhibit podocyte apoptosis, promote angiogenesis and cell survival, and reduce protein in rats with diabetic nephropathy. abilities as their parent cells [140]. Exosomes derived from adipose tissue stem cells (ADSCs) (ADSCs-Exo) can inhibit mTOR activation by upregulating miP-486 and suppressing Smad1 expression, leading to increased autophagy and reduced podocyte apoptosis, alleviating spontaneous diabetes mellitus and improving symptoms diabetic nephropathy [141]. It can be seen that exosomes have broad prospects for clinical application for the prevention of diabetic kidney damage [142].

### 4.2 Exosomes and diabetic retinopathy (DR)

DR is a microvascular complication of diabetes and a major cause of vision loss in adults [143]. Diabetic peripheral vascular disease with ocular complications accounted for 41% of severe cases of DR.

Studies have shown that cytokines (RANTES and Ang-2) in plasma exosomes are involved in modulating the course and prognosis of DR [144]. Exosomes secreted by pancreatic β cells contain miR-15a, which can induce human Müller cells to overexpress miR-15a, and then target Akt3 to cause oxidative stress, apoptosis, and retinal damage [105]. Exosomal miR-222 expression levels released from MSCs isolated from diabetic adipose tissue were inversely correlated with retinal repair [145]. The activation of the classical complement pathway by plasma exosomes containing IgG is also involved in the progression of DR [143].

### 4.3 Exosomes and diabetic neuropathy

Studies have shown that exosomes secreted by M-MSC can repair damaged neurons and astrocytes and reverse their dysfunction, indicating that exosomes may be an ideal treatment for diabetic nerve injury [146]. Enriched medium (enriched medium, EE) stimulates the activation of miR-146a in exosomes secreted by endogenous M-MSCs, which has an anti-inflammatory effect on damaged astrocytes in the brain of diabetic rats and may prevent cognitive impairment caused by diabetes [147].

### 4.4 Exosomes and diabetic skeletal muscle and bone metabolic lesions

Skeletal muscle exosomes regulate skeletal muscle homeostasis in a state of insulin resistance caused by a high-fat diet through a similar paracrine transmission method, and they can be absorbed by the pancreas [148]; miR-16 in exosomes is involved in high-fat diet Induced changes in the proliferation of MIN6B1 cells and islets and regulation of the Ptch1 gene, thereby participating in the development of the pancreas [60]. Bone marrow-derived exosomal miRNAs differ in quantity, type, and expression level, and exosomal miRNA profiles targeting insulin secretion and insulin signaling pathways are altered under T2DM conditions, in which alterations in the Wnt signaling pathway are key to bone metabolism [149].

### 4.5 Exosomes and cardiovascular complications of diabetes

Insulin is essential for cardiac contractility, growth, and metabolism, thus impaired insulin signaling plays a key role in diabetic cardiovascular complications [150]. Diabetic cardiovascular complications are the main cause of disability and death in diabetic patients. These complications are closely related to insulin resistance and dyslipidemia. Since different types of cardiac cells secrete their own cardiac exosomes, the academic community began to speculate that exosomes may be involved in the pathophysiology of cardiovascular diseases including diabetic cardiomyopathy (DCM) [151]. In the future, exosomes may be used to treat diabetic myocardial damage to some extent [152].

In the early stages of diabetes, hyperglycemia can lead to endothelial and microvascular dysfunction [153,154]. Dysregulation of myocardial angiogenesis has been suggested to be a key cause of diabetic cardiovascular disease, and cardiac endothelial cells play a key role in cardiomyocyte function and structure [[155], [156], [157], [158], [159]]. Exosomes contain pathogenic factors of diabetic atherosclerosis [160]. Serum exosomes from diabetic db/db mice were ingested by normal mouse aortic endothelial cells, and severe endothelial dysfunction occurred, which was caused by the transfer of arginase-1 (arginase 1) by serum exosomes to endothelial cells [161]. Mammalian sterile 20-like kinase 1 (MST1)-rich exosomes released from cardiac microvascular endothelial cells (CMECs) have pleiotropic effects in inhibiting autophagy and can promote cellular Apoptosis, inhibition of cellular glucose metabolism [162]. miRNAs (such as miR-214, miR-143/145) encapsulated in exosomes secreted by vascular endothelial cells also play an important role in angiogenesis and anti-atherosclerosis [163,164].

Cardiomyocyte-derived exosomes, known as cardiosomes, contain variable amounts of nucleic acids, proteins, and lipids, which can be transferred to adjacent cardiac endothelial cells and regulate their function [[165], [166], [167], [168], [169]]. Cardiosomes wrap miR-455, miR-29b, miR-323–5p and miR-466, these miRNAs can bind to metalloproteinase-9 (matrix metalloprotease 9, MMP9) and down-regulate its expression, reduce myocardial fibrosis, inhibit Cardiomyocyte decoupling, thereby promoting myocardial regeneration [170]. Cardiosomes can also regulate glucose transport in endothelial cells [152]. The levels of miR-1 and miR-133a were higher in lipid-preconditioned cardiosomes, and these miRNAs were positively correlated with diabetic myocardial damage [171]. Cardiosome-rich HSP70 activates the cardioprotective signaling pathway induced by ERK1/2 and HSP27 in cardiomyocytes; when T2DM occurs, although the expression of cardiosome HSP70 still increases, it loses its protective effect on the heart [172]. Diabetic cardiosomes were used to intervene cardiomyocytes under hypoxia-reoxygenation, exacerbating cell death [172]. It shows that diabetic myocardial vascular damage may be caused by anti-angiogenic exosomes secreted by cardiomyocytes [166].

In addition, the increased angiogenesis of vasa vasrum (VV) can promote the rupture of T2DM atherosclerotic plaque, and ADEs participate in the promotion of plaque aggravation and plaque damage, partly through the induction of VV angiogenesis, and partly through the above mentioned exosomes to aggravate diabetic atherosclerosis [173].

Therefore, for diabetic cardiovascular disease, exosomes can not only serve as potential biomarkers, but also have therapeutic effects, and can be used as targets or drugs to reverse the impaired insulin signaling.

## 5 Conclusion

**Fig. 2:**
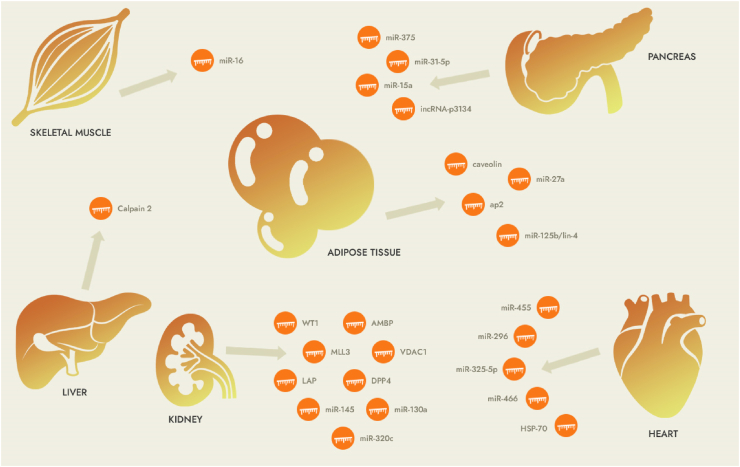
Exosomes as mediators of related organs in diabetes.

Due to the complexity of clinical manifestations of different types of diabetes, sometimes relying on the current laboratory methods cannot be identified in time. Therefore, it is urgent to find a marker that can not only reflect the pathophysiological characteristics or disease progression in real time, but also be simple, cheap, and easy to operate [174]. Perhaps in the future, exosomes will be able to meet this new requirement for diabetes biological markers [20]. People can monitor physiological and pathological changes by analyzing the contents of lipids, proteins, nucleic acids, etc. in exosomes (Fig. 2, Table 1).

**Table 1:** The role of exosomes in the diagnosis and treatment of diabetes and its complications.

Source of exosomes	Bioactive factors	Disease model	Application	References
HepG2	Calpain 2	High-glucose conditions	Treatment	[29]
Mouse skeletal muscle	miR-16	HPD	Treatment	[60]
Mouse adipocyte	Caveolin 1	Obesity	Treatment	[34]
Human/mouse adipocyte	miR-125b/lin-4	Obesity	Diagnosis	[39]
Mouse adipocyte	miR-27a	Obesity	Treatment	[41]
Human adipose-derived stem cells	MALAT1	Obesity	Diagnosis	[42]
Mouse serum	Adiponectin	Obesity	Diagnosis	[45]
Mouse adipocyte	ap2	Obesity	Treatment	[47]
3T3-L1	Shh	High-glucose conditions	Treatment	[48]
M-MSCs	miR-29b-3p	Senescence	Treatment	[53]
Mouse pancreas	miR-375	Normal	Diagnosis/treatment	[[67], [68], [69], [70]]
Human urine	miR-130a	DN	Diagnosis	[67]
Macrophage	miR-155	Obesity	Diagnosis/treatment	[71]
PBMC	miR-126	T2DM	Diagnosis	[72]
Mouse plasma	miR-27b-3p	Obesity	Diagnosis	[73]
Human/Mouse pancreatic β cells	miR-21–5p	T1DM	Diagnosis	[97,98]
Human plasma	miR-25–3p	T1DM	Treatment	[99]
Human pancreatic β cells	miR-15a	T2DM	Diagnosis	[105]
Human plasma	miR-126	Prediabetes	Diagnosis	[106]
Human/Mouse plasma	miR-192/193	Prediabetes	Diagnosis	[107]
Human pancreatic β cells	lncRNA-p3134	T2DM	Diagnosis	[109]
Human urine	miR-145	DN	Diagnosis	[117]
Human endothelial cells	PAPP-A	GDM	Diagnosis	[129]
Mouse serum	Arginase 1	db/db	Diagnosis	[142]
Mouse cardiac microvascular endothelial cells	MST1	T1DM (STZ + mouse)	Treatment	[143]
Human/Mouse endothelial cells	miR-143/145	Lipid loaded	Treatment	[144]
HMEC-1	miR-214	Senescence	Treatment	[145]
Mouse cardiomyocyte	mir-466	db/db	Treatment	[151]
HL-1	miR-133a	Lipid-loaded	Diagnosis	[152]
Rat cardiomyocyte	HSP70	Gotokakizaki	Treatment	[153]
Human urine	WT1	T1DM	Diagnosis	[157]
Human urine	VDAC1	DN	Diagnosis	[158]
Human urine	LAP	T2DM	Diagnosis	[159]
Human urine	miR-320c	DN	Diagnosis	[161]
Adipose-derived stem cells	miR-486	DN	Diagnosis	[165]
Human plasma	RANTES	DR	Diagnosis	[168]
Rabbit adipose MSCs	miR-222	T1DM (STZ + rabbit)	Treatment	[169]
M-MSCs	miR-146a	T1DM (STZ + rat)	Treatment	[171]

AbbreviationM-MSCs: marrow mesenchymal stem cells; MSCs: mesenchymal stem cells; PBMC: peripheral blood mononuclear cell; T1DM: type 1 diabetes mellitus; T2DM: type 2 diabetes mellitus; GDM: gestational diabetes mellitus; DN: diabetic nephropathy; DR: diabetic ret-inopathy; HPD: high palmitate diet.

At the same time, exosomes have unique advantages as a natural endogenous drug carrier: they have good immunocompatibility, low immunogenicity, skillfully avoid the rapid clearance of monocytes and macrophages, and prolong the life of exosomes. peripheral circulation shows greater stability in the blood, which increases efficiency [175,176]; it is widespread and can even cross the complete blood-brain barrier [177]; its diameter is just right for use Enhanced Permeability and Retention Effect (EPR) selectively extravasates into certain specific tissues;

Exosomes can also deliver drugs to specific tissues or organs [[178], [179], [180], [181]]. Therefore, exosomes may overcome the current difficulties with the encapsulation of siRNA-based nucleic acid preparations and enable the widespread use of nucleic acid preparations in clinical practice in the future. Currently known miRNAs in exosomes that can be used as targets include: miR-27a, miR-155, miR-143/145, miR-16 associated with improved miR-222, miR-146a, miR-25-3r, miR-16-5r, etc. associated with DM1, miR-455, miR-296 associated with DM2, miR-323–5p, miR-466 [151]. Exosome research has a positive impact on the development of future diabetes drugs, especially targeted therapies (see summary in Table 1 and Fig. 2).

Current research on the role of exosomes carrying noncoding RNAs in diabetes is in its early stages, particularly with regards to lncRNAs, circRNAs, and PIWI-interacting RNAs. There is also a lack of clear characterizations and markers for exosomes from different cell types, making it challenging to determine the source of exosomes in circulation [182,183]. Although the use of exosomes as drug carriers has been widely studied, there are still challenges in developing exosome-based drug delivery systems, such as efficient cargo encapsulation, selecting appropriate exosome-originating cells, drug loading methods, and modifying exosome surfaces [[184], [185], [186], [187], [188]]. However, exosomal miRNAs and lncRNAs have been shown to play a role in modulating the progression of diabetes, including affecting metabolic and insulin signals in target tissues, cell viability, and pancreatic cell inflammation.

Thus, people's deep understanding of the mechanism of exosome production, regulation of secretion or uptake provides a better understanding of the pathophysiological mechanism of diabetes, as well as for the development of more methods for diagnosing and treating diabetes and its complications in the future are provided.

## Funding

This work was supported by the Bashkir State Medical University Strategic Academic Leadership Program (PRIORITY-2030).

## Author contributions

Albert Sufianov and Andrey Kostin conceptualized and designed the study. All authors have participated in the acquisition, analysis and interpretation of the data. Sema Begliarzade and Valentin Kudriashov has drafted the manuscript. Tatiana Ilyasova, Yanchao Liang and Albert Mukhamedzyanov contributed to the critical revisions of the manuscript. Ozal Beylerli supervised the research. All authors agreed on the journal to which the article would be submitted, gave the final approval for the version to be published, and agreed to be accountable for all aspects of the work.

## Declaration of competing interest

The authors declare that no conflicts of interest exist.
